# Identifying the Effect of Celastrol Against Ovarian Cancer With Network Pharmacology and *In Vitro* Experiments

**DOI:** 10.3389/fphar.2022.739478

**Published:** 2022-03-18

**Authors:** Xuan Wang, Qiong Liu, Sisi Wu, Nana Xu, Hua Li, Aihua Feng

**Affiliations:** Yichang Central People’s Hospital, The First Clinical Medical College of Three Gorges University, Yichang, China

**Keywords:** celastrol, ovarian cancer, network pharmacology, molecular docking, inflammatory responses

## Abstract

**Aim:** We aimed to reveal the function of celastrol in the treatment of ovarian cancer using network pharmacology and molecular docking.

**Background:** Ovarian cancer is a growth of cells that forms in the ovaries. Celastrol is a useful bioactive compound derived from the root of the thunder god vine.

**Method:** Celastrol and ovarian cancer targets were determined by analyzing datasets. Protein–protein interaction (PPI) networks were obtained with network pharmacology. Then, Gene Ontology (GO) and Kyoto Encyclopedia of Genes and Genomes (KEGG) analyses were performed. Molecular docking using SWISS-MODEL, CB-Dock and Discovery Studio was conducted. A methylthiazolyltetrazolium bromide (MTT) assay was performed to evaluate cell proliferation. Cell apoptosis and cell cycle were measured with a fluorescence assay. Reverse transcription PCR (RT-PCR) and Western blot were performed to measure the expression of core targets.

**Result:** Celastrol possessed 29 potential targets, while ovarian cancer possessed 471 potential targets. The core PPI network contained 163 nodes and 4,483 edges. The biological processes identified in the GO analysis indicated that the targets were related with the cellular response to DNA damage stimulus, DNA recombination, and cell proliferation, among other processes. The KEGG analysis indicated that the pathways were related with the cell cycle, viral carcinogenesis, and MAPK signaling pathway, among others. The three core targets shared between the core PPI network and celastrol targets were MYC, CDC37, and FN1. Celastrol directly combined with the targets according to the results from CB-Dock and Discovery Studio. Celastrol inhibited ovarian cancer cell proliferation and promoted ovarian cancer cell apoptosis in a dose-dependent manner. RT-PCR and Western blot analyses showed that celastrol inhibited core target expression. In addition, celastrol also influenced the related inflammatory signaling pathways in ovarian cancer cells.

**Conclusion:** Celastrol exerts effective antitumor activity toward ovarian cancer. Celastrol regulated cell proliferation, DNA repair and replication, apoptotic processes, and inflammatory responses in ovarian cancer cells.

## Introduction

Ovarian cancer is a growth of cells that forms in the ovaries (La Vecchia, 2017). New cases of ovarian cancer are estimated to range from 5 to 9.4 per 100,000 annually. The annual prevalence of ovarian cancer is 33 per 100,000 in women over 50 years old. The symptoms of ovarian cancer are often undetected in the initial stages of ovarian cancer (Ebell et al., 2016). Symptoms including pelvic pain, bloating, and loss of appetite become more noticeable as the cancer progresses. The risk factors for ovarian cancer are environmental factors, gene mutations, fertility medication, and alcohol consumption (Rooth, 2013). The management of ovarian cancer involves surgery, chemotherapy, radiotherapy, hormone therapy, and immunotherapy (Orr and Edwards, 2018). Due to the lack of early detection, ovarian cancer usually has a poor prognosis, with a 46% 5-year survival rate. In the context of the poor prognosis of patients receiving current treatments, studies exploring novel therapies for ovarian cancer are crucial.

Celastrol is a beneficial bioactive compound derived from the root of the thunder god vine (*Tripterygium wilfordii* Hook F, TwHF) (Chen et al., 2018). It is classified as a pentacyclic triterpenoid from the quinone methide family. Celastrol has been used to treat joint pain, edema, and fever. It has also been discovered to possess therapeutic properties against obesity, rheumatoid arthritis, neurodegenerative diseases, and cancer (Liu et al., 2015; Li and Hao, 2019; Song et al., 2019). In *in vivo* and *in vitro* experiments, celastrol exerts anti-inflammatory, antioxidant, and anticancer effects (Cascão et al., 2017). In the treatment of cancer, celastrol induces cancer cell death, inhibits angiogenesis, and enhances sensitivity to radiotherapy (Kashyap et al., 2018). Recent studies have also investigated the effect of celastrol on ovarian cancer. Li et al. found that celastrol inhibited the proliferation and migration of ovarian cancer cells by inducing G2/M arrest in the cell cycle (Li et al., 2019). Xu et al. reported that celastrol induces apoptosis by increasing intracellular reactive oxygen species production (Xu et al., 2019). According to Wang et al., celastrol suppresses the invasion and migration of ovarian cancer cells by inhibiting the NF-κB signaling pathway (Wang et al., 2017). However, researchers have not provided a holistic view of the effects of celastrol on ovarian cancer.

Therefore, we aimed to reveal the function of celastrol in the treatment of ovarian cancer using *in vitro* and *in vivo* experiments. Firstly, we determined the targets of celastrol and ovarian cancer. Two PPI networks related to celastrol and ovarian cancer targets were constructed. The two PPI networks were merged, and a core PPI network was obtained based on network topological features. Secondly, GO and KEGG analyses revealed the related signaling pathways and biological processes (BPs). Thirdly, molecular docking was conducted to predict the binding affinity between celastrol and key molecules related to ovarian cancer. At last, we determined the effects of celastrol *in vitro*. Methylthiazolyltetrazolium bromide (MTT) assay, reverse transcription PCR, flow cytometry, and Western blot were performed to validate the function of celastrol. This research could provide novel insights into and a meaningful analysis of the therapeutic effect of celastrol on ovarian cancer.

## Methods

### Determination of Ovarian Cancer and Celastrol Targets

Four related databases were selected to determine the targets of ovarian cancer. The databases included the Genetic Association Database (GAD), Therapeutic Target Database (TTD), PharmGKB, and Online Mendelian Inheritance in Man (OMIM). The GAD database is a useful dataset to discover the correlations between genes and diseases. TTD is a powerful dataset for exploring nucleic acid targets and therapeutic proteins. PharmGKB is an online database of human genetic variation in drug response. The OMIM database provides information on human genes and genetic diseases. The targets of celastrol were evaluated using the Traditional Chinese Medicine Systems Pharmacology Database and Analysis Platform (TCMSP) database. TCMSP is a pharmacology platform that includes targets, chemicals, and drug-target networks.

### Construction of PPI Networks

The BisoGenet plugin in Cytoscape was used to establish PPI networks. Six related PPI databases were analyzed using the BisoGenet plugin, including the Biomolecular Interaction Network Database (BIND), Biological General Repository for Interaction Datasets (BioGRID), Database of Interacting Proteins (DIP), Human Protein Reference Database (HPRD), IntAct Molecular Interaction Database (IntAct), and Molecular INTeraction Database (MINT). In addition, Cytoscape software was conducted to combine the PPI networks of celastrol and ovarian cancer. Cytoscape software was conducted to conceive the networks.

### Analysis of Network Topological Features

The CytoNCA plugin was conducted to analyze network topological features. We used six related parameters to analyze the topological features of the interaction network. The parameters included betweenness centrality (BC), closeness centrality (CC), degree centrality (DC), Eigenvector centrality (EC), local average connectivity (LAC), and network centrality (NC).

### Gene Ontology and Kyoto Encyclopedia of Genes and Genomes Analyses

The Database for Annotation, Visualization, and Integrated Discovery (DAVID) was used to perform functional enrichment analyses, including GO and KEGG analyses. GO analysis was performed to explore BPs. KEGG analysis identified the signaling pathways related to genes, drugs, and diseases.

### SWISS-MODEL

In this study, we used SWISS-MODEL to construct the tertiary structure of core targets. SWISS-MODEL is a homology modeling server that automatically builds the tertiary structure of proteins. Ramachandran plots and local quality estimate graphs of the three targets were drawn using SWISS-MODEL.

### CB-Dock

In this study, we used CB-Dock to perform molecular docking. CB-Dock is a protein-ligand docking method designed to identify binding sites, analyze center and size, and conduct molecular docking. It facilitates docking procedures and increases the accuracy of molecular docking. Cavity-focused docking increases the accuracy and hit ratio with blind docking. Cavity size and Vina score were evaluated using CB-Dock. The “spacefill” and “cartoon” parameters were set for the ligand and receptor, respectively. “Element” and “chain” were used for ligands and colored receptors.

### Discovery Studio

Discovery Studio is a powerful software to simulate small molecules and macromolecules. The software includes simulations, ligand design, structure-based design, macromolecule engineering, and other processes. The simulation area has molecular dynamics, molecular mechanics, and quantum mechanics. Ligand design includes tools for enumerating molecular libraries and library optimization. Pharmacophore model includes virtual screening, validation, and creation. Discovery Studio could drive practical explorations to optimize biochemical potency and characteristics.

### Cells Lines and Reagents

The human A2780 ovarian cancer line was performed in our experiments. Cells were cultured in RPMI 1640 medium containing 10% fetal bovine serum (FBS), streptomycin (100 ng/ml), and penicillin (100 U/ml). The parameters of the incubator were 5% CO_2_ and 37°C. Celastrol was obtained from Sigma–Aldrich. Annexin V FITC, and PI were obtained from Abcam as part of a cell apoptosis kit (ab273273). Antibodies against β-actin (8457), c-myc (9402), CDC37 (3604), FN1 (26836), phospho-p44/42 MAPK (Erk1/2) (4370), phospho-NF-κB p65 (Ser536) (3033), phospho-Akt (Thr308) (13038), BAX (2774), Bcl-2 (15,071), phospho-JNK (9251), and phospho-p38 MAPK (4511), HIF-1α (36169) were obtained from Cell Signaling Technology (CST).

### Cell Proliferation Assay

A MTT assay was conducted to measure the effect of celastrol on cell proliferation. A2780 cells were cultured with different celastrol concentrations ranging from 100 nM to 1 µM, while the control group was cultured with RPMI 1640 medium. After an incubation for 96 h, the medium was removed, and the cells were incubated with the MTT solution for 4 h at 37°C. The absorbance of the wells was measured with a microplate reader at 570 nm.

### Cell Apoptosis and Cell Cycle Analysis

A cell apoptosis kit (556547, BD Biosciences) was used to measure apoptosis. A2780 cells were harvested and washed two times with phosphate-buffered saline (PBS). Then, cells were stained with Annexin V-FITC and PI. After a 15 min incubation, the cells were measured with flow cytometry. The percentage of apoptotic cells was calculated.

A cell cycle kit (C1052, Beyotime) was used to detect the cell cycle. Cells were harvested and washed two times with PBS. Then, the cells were fixed with 70% ethanol for 2 h. After washing with PBS, the cells were stained with a mixed solution containing PI and RNase A for 30 min at 37°C. Cell cycle analyses were detected by flow cytometry. The percentage of G1, S, and G2 phases were measured.

### Quantitative RT-PCR

Total RNA was extracted with TRIzol (Invitrogen, Grand Island, NY, United States). One microgram of RNA was reverse transcribed to cDNAs. The primers used in our study were as follows: human myc (forward: 5′-aac​aca​caa​cgt​ctt​gga​gc-3′, reverse: 5′-gca​caa​gag​ttc​cgt​agc​tg-3′), CDC37 (forward: 5′-agg​tgg​agg​aga​aat​gtg​ca-3′, reverse: 5′-ctt​cat​ggc​ctt​ctc​gat​gc-3′), FN1 (forward: 5′-ccc​cat​tcc​agg​aca​ctt​ct-3′, reverse: 5′-tgc​ctc​cac​tat​gac​gtt​gt-3′), and β-actin (forward: 5′-cat​gga​atc​ctg​tgg​cat​cc -3′, reverse: 5′-cac​aca​gag​tac​ttg​cgc​tc -3′).

### Western Blot Analysis

Cells were lysed in RIPA buffer with 10% phenylmethylsulfonyl fluoride (PMSF) about 15 min. The lysates were centrifuged at 14,000 *g* for 15 min. After centrifugation, the supernatants were collected and boiled for 15 min. Twelve percent sodium dodecyl sulfate polyacrylamide gel electrophoresis (SDS-PAGE) gels separated the proteins and transferred them to a nitrocellulose membrane. The parameters used to transfer proteins were 120 V for 120 min. After blocking, the dilution of primary CST antibodies was 1:1,000. The membranes were incubated with primary antibodies overnight with 4°C. On the second day, the membrane was incubated with horseradish peroxidase-conjugated secondary antibodies. Then, the membrane was detected with detection reagents.

### Statistical Analysis

The data are presented as the means ± standard deviations (SD). GraphPad Prism software was used to draw graphs. The comparison among groups were tested using one-way analysis of variance (ANOVA). The Student's t-test was used to analyze the differences between two groups. *p* < 0.05 was considered statistically significant.

## Results

### Determination of Celastrol and Ovarian Cancer Targets

**FIGURE 1:**
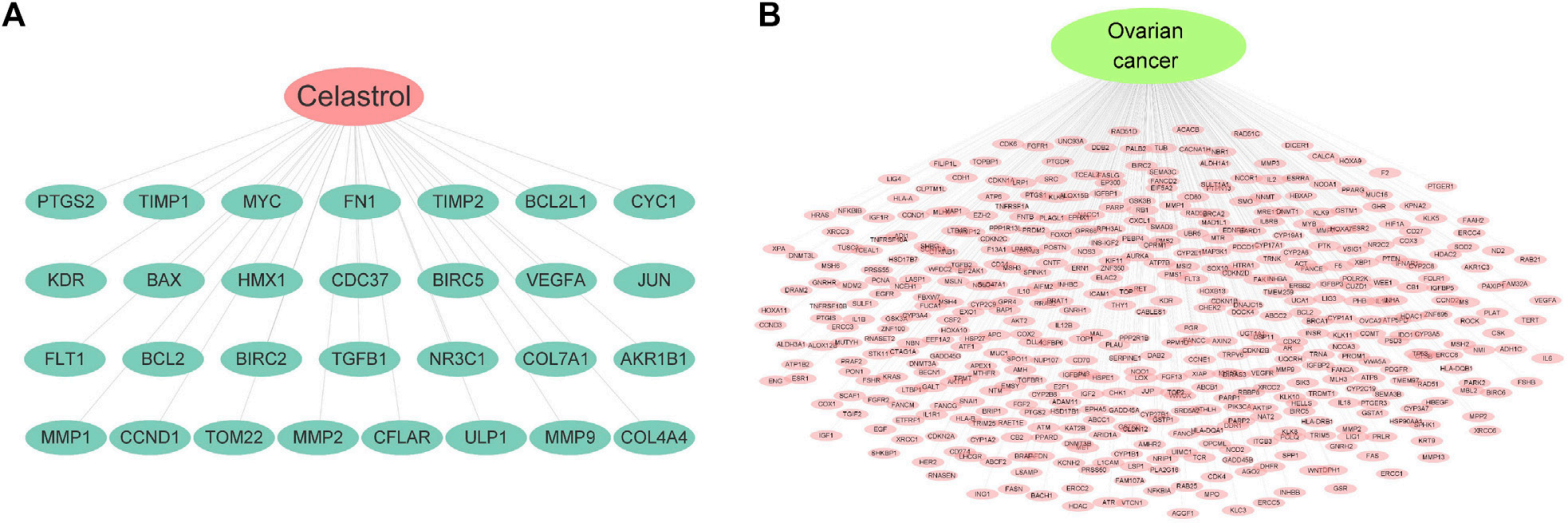
Potential targets of celastrol and ovarian cancer. **(A)** Targets of celastrol. The pink node represents celastrol, while green nodes show the related targets. **(B)** Targets of ovarian cancer. The green node represents ovarian cancer, while the pink nodes show the related targets.

In this study, 29 potential targets of celastrol were identified with the TCMSP database. The potential targets of celastrol contain CYC1, HMX1, TOM22, ULP1, MMP2, AKR1B1, and BAX, among others. Figure 1A indicates the potential targets of celastrol. In addition, 471 potential targets in ovarian cancer were identified using four related databases. The potential targets of ovarian cancer include ATP6, BRAF, COX2, EGFR, and IL6, among others. Figure 1B indicates the potential targets of ovarian cancer.

### PPI Network Construction

**FIGURE 2:**
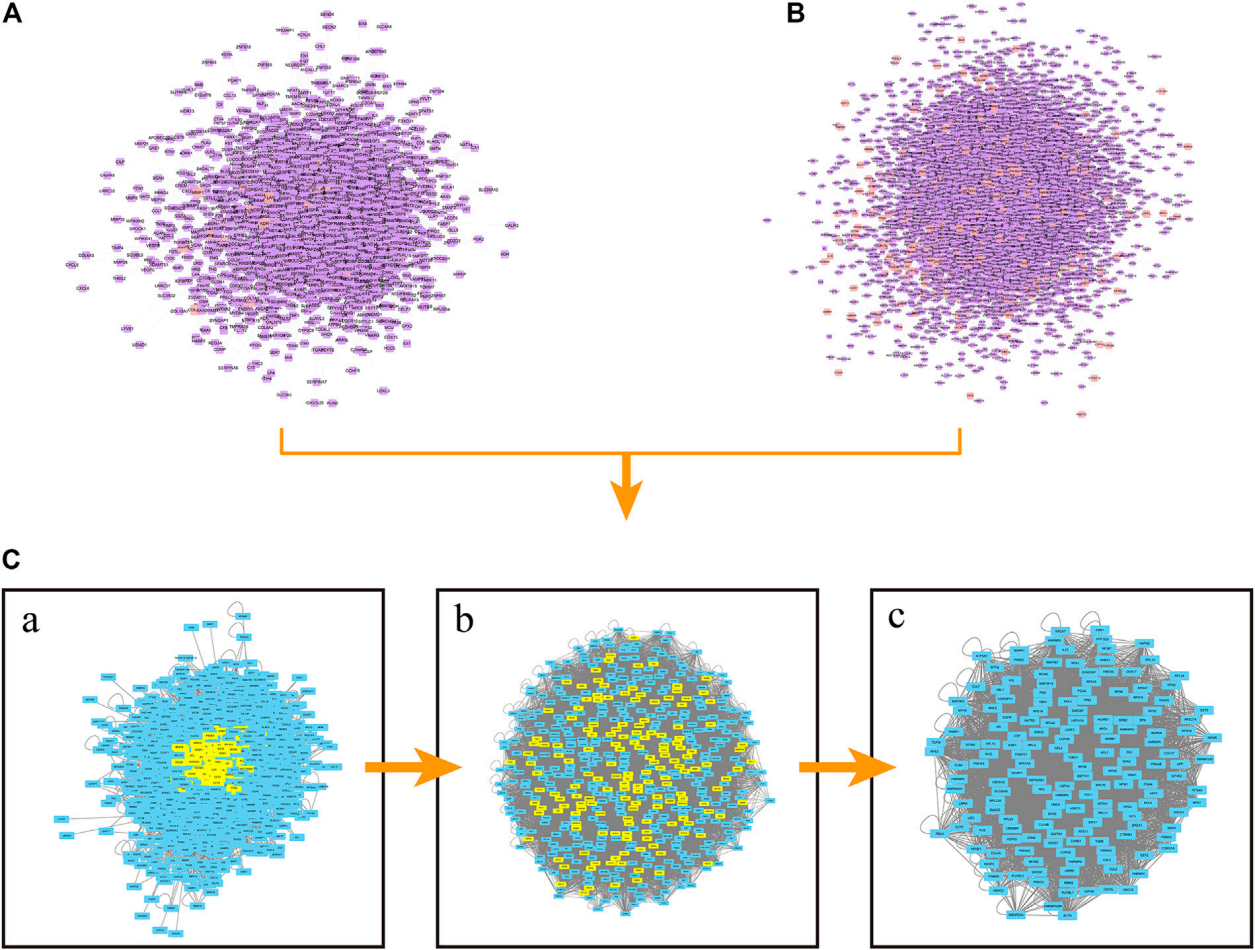
PPI network construction. **(A)** PPI network of celastrol. Orange square nodes represent the celastrol targets. Purple square nodes represent proteins interacting with celastrol targets. **(B)** PPI network of ovarian cancer. Orange square nodes represent the ovarian cancer targets. Purple square nodes represent proteins interacting with ovarian cancer targets. **[C(a)]** Merged PPI network. **[C(b)]** PPI network filtered based on the parameter DC > 67. **[C(c)]** Core PPI network.

PPI networks were constructed with Cytoscape software to reveal the correlations of proteins between celastrol and ovarian cancer. Figure 2A shows the PPI network of celastrol. The PPI network of celastrol contained 2,500 nodes and 59,307 edges. Figure 2B shows the PPI network of ovarian cancer. The PPI network of ovarian cancer comprised 8,316 nodes and 1,92,286 edges. Furthermore, we merged the PPI networks of celastrol and ovarian cancer to obtain a new PPI network. Figure 2C(a) shows the merged PPI network. The merged PPI network included 2,212 nodes and 56,260 edges. After obtaining a merged PPI network, we performed a topological feature analysis to generate a core PPI network. The parameter DC > 67 was first used to filter the data (Figure 2C(b)). Then, six parameters, BC > 0.000743, CC > 0.531, DC > 66, EC > 0.030049726, LAC > 19.25, NC > 21.78499906, were applied to obtain the core PPI network. As shown in Figure 2C(c), the core PPI network contained 163 nodes and 4,883 edges. The nodes in the core PPI network included MCM7, NFKB2, FLNA, UBC, and ESR1.

### GO and KEGG Analyses

**FIGURE 3:**
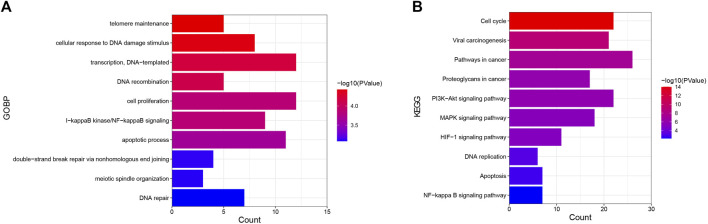
GO and KEGG analyses of targets of celastrol involved in its therapeutic effect on ovarian cancer. **(A)** GO analysis. The *Y*-axis shows the enriched GO items of target genes. The *X*-axis shows the gene counts for the items. The color indicates the *p* value. **(B)** KEGG analysis. The *Y*-axis shows the enriched KEGG pathways for target genes. The *X*-axis shows the gene counts for the items. The color indicates the *p* value.

One hundred sixty-three nodes were obtained in the core PPI network. These nodes were subjected to GO and KEGG analyses to reveal the function of celastrol. The BPs identified in the GO analysis indicated that the node proteins were related with telomere maintenance, cellular response to DNA damage stimulus, DNA recombination, cell proliferation, I-kappaB kinase/NF-kappaB signaling, apoptotic process, and other processes (Figure 3A). The KEGG analysis indicated that the pathways were enriched in the cell cycle, viral carcinogenesis, pathways in cancer, proteoglycans in cancer, PI3K-Akt signaling pathway, MAPK signaling pathway, HTLV-I infection, HIF-1 signaling pathway, DNA replication, etc. (Figure 3B).

### The Prediction of the Tertiary Structures of Core Targets

**FIGURE 4:**
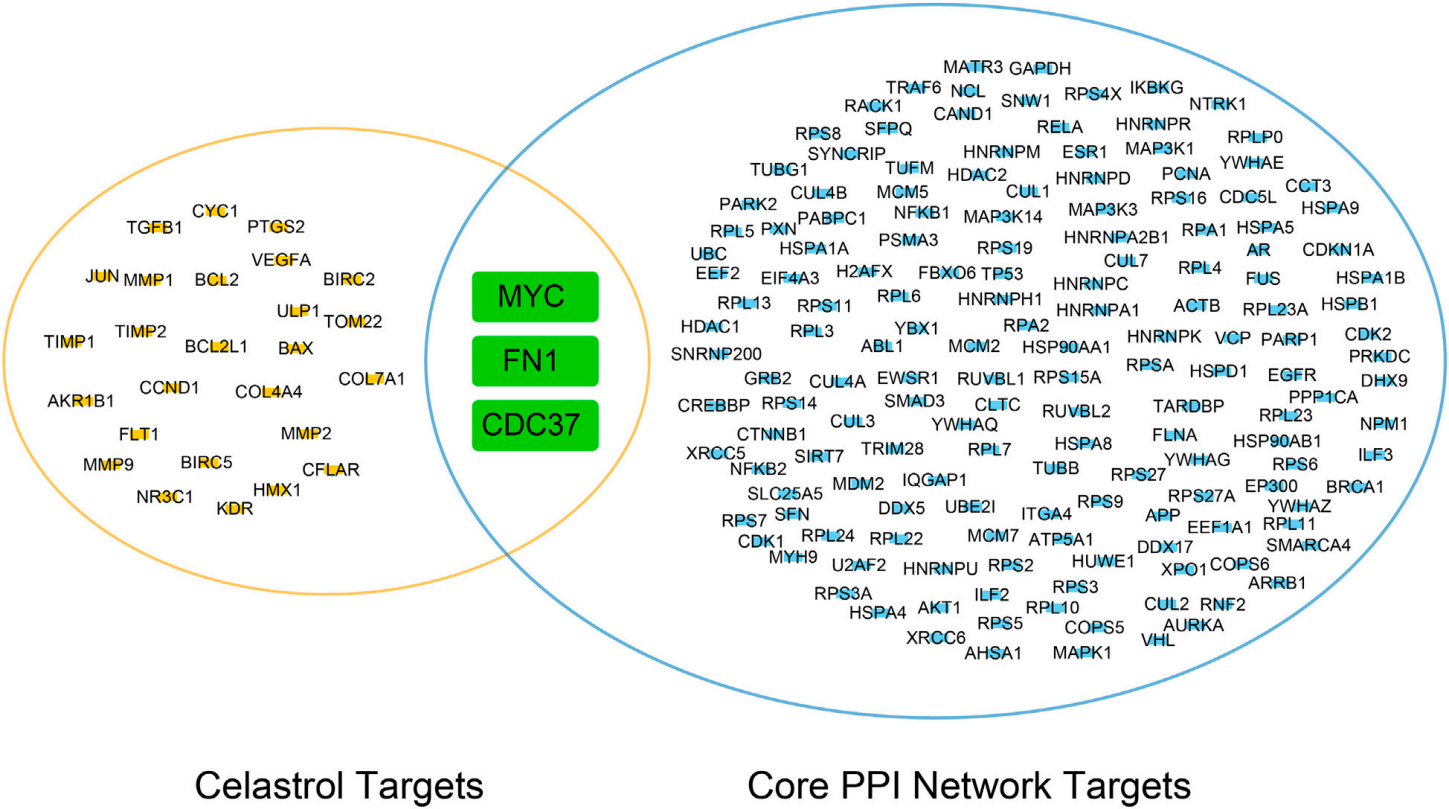
Venn diagram of the core PPI network and celastrol targets. The core targets were MYC, FN1, and CDC37.

**FIGURE 5:**
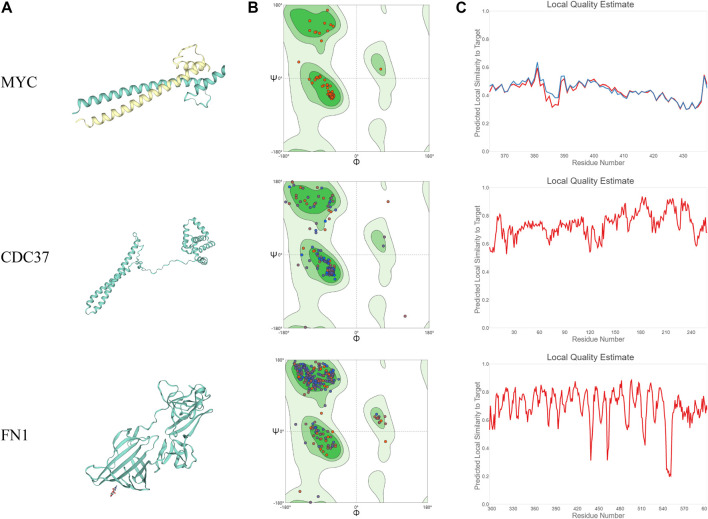
Analysis of the MYC, CDC37, and FN1 protein structures. **(A)** 3D visualization of the structures of MYC, CDC37, and FN1. **(B)** Ramachandran plots of MYC, CDC37, and FN1. **(C)** The local quality estimate graphs of MYC, CDC37, and FN1.

In this study, we merged the core PPI network and celastrol targets to obtain the core targets. The three core targets were MYC, CDC37, and FN1 (Figure 4). The sequences of these three proteins were searched in the Protein Data Bank (PDB). Then, the tertiary structures of these three targets were built using SWISS-MODEL, a fully automated protein structure homology-modeling server. Figure 5A shows the three-dimensional (3D) structures of MYC, CDC37, and FN1. The Ramachandran plots in Figure 5B show that these three proteins had a high percentage of protein residues in the favorable region. Figure 5C indicates the predicted local similarity of these three selected protein sequences to target sequences.

**FIGURE 6:**
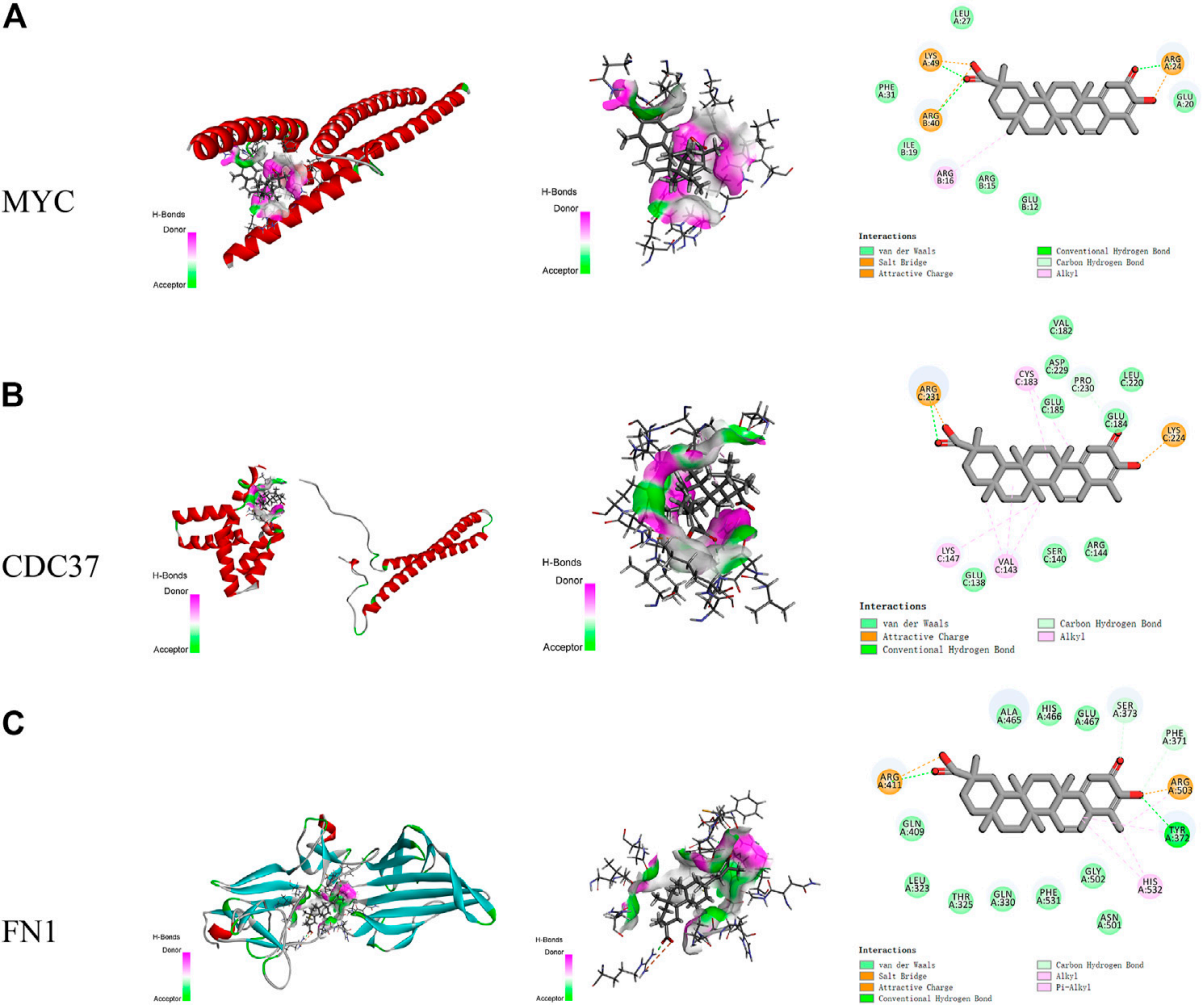
The interactions between celastrol and its core targets. **(A)** The interaction between celastrol and MYC. **(B)** The interaction between celastrol and CDC37. **(C)** The interaction between celastrol and FN1. In H-bonds, pink indicates the donor, while green indicates the acceptor. In the interactions, green indicates van der Waals interactions, light green indicates carbon-hydrogen bonds, dark green indicates conventional hydrogen bonds, yellow indicates salt bridges and attractive charges, and pink indicates alkyl and pi-alkyl interactions.

Discovery Studio was used to identify the interactions between celastrol and amino acids in the core targets (Figure 6). The amino acids in MYC that interact with celastrol include LYS A:49, ARG B:40, and ARG A:24, among others. The distance-dependent interactions between MYC and celastrol had van der Waals interactions, attractive charges, salt bridges, conventional hydrogen bonds, carbon-hydrogen bonds, and alkyl bonds. The amino acids in CDC37 that interact with celastrol include ARG C:231, CYS C:183, and PRO C:230, among others. The distance-dependent interactions between CDC37 and celastrol included van der Waals interactions, attractive charges, conventional hydrogen bonds,carbon-hydrogen bonds, and alkyl interactions. The amino acids in FN1 that interact with celastrol include ARG A:411, ARG A:503, HIS A:532, and TYR A:372. The distance-dependent interactions between FN1 and celastrol included van der Waals interactions, salt bridges, attractive charges, conventional hydrogen bonds, carbon hydrogen bonds, alkyl bonds, and pi-alkyl interactions.

The top five Vina scores of celastrol with the core targets were acquired using CB-Dock. The lowest Vina scores showed the most stable targets binding to celastrol. In the interaction of MYC with celastrol, the lowest Vina score was −7.1 kcal/mol, while the cavity size was 65 Å^3^. In the interaction of CDC37 with celastrol, the lowest Vina score was −7.6 kcal/mol, while the cavity size was 79 Å^3^. In the interaction of FN1 with celastrol, the lowest Vina score was −9.2 kcal/mol, while the cavity size was 270 Å^3^.

### Celastrol Inhibited Ovarian Cancer Cell Proliferation

**FIGURE 7:**
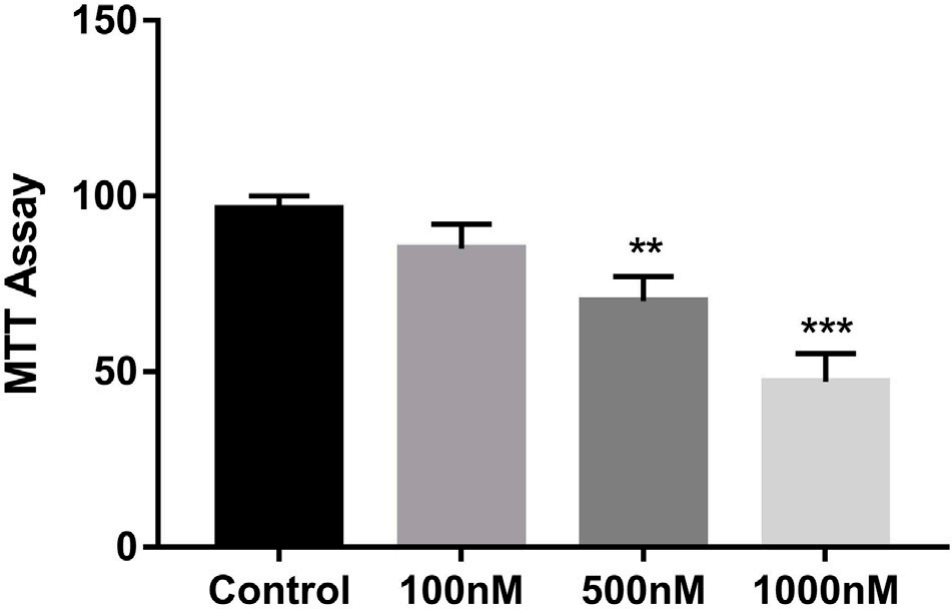
Celastrol inhibited ovarian cancer cell proliferation. The MTT assay was used to measure cell growth. Compared with the control group, **p < 0.01, ***p < 0.001.

We used the A2780 cell line to test the effect of celastrol on the proliferation of ovarian cancer cells. Different concentrations of celastrol ranging from 100 nM to 1 µM (100, 500, and 1,000 nM) were applied. As shown in Figure 7, celastrol suppressed the proliferation of ovarian cancer cell lines in a dose-dependent manner.

### Effect of Celastrol on Apoptosis and the Cell Cycle in Ovarian Cancer

**FIGURE 8:**
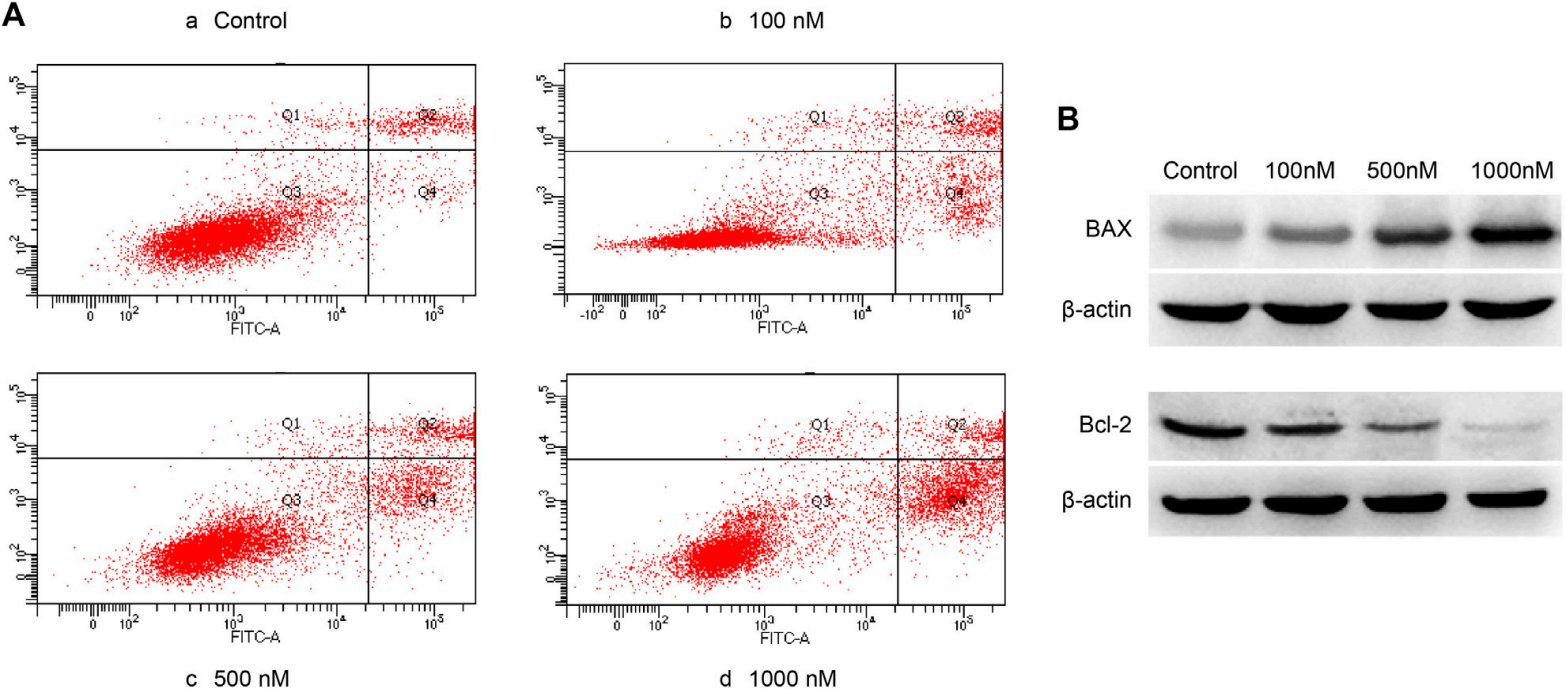
Celastrol promoted ovarian cancer cell apoptosis. **(A)** The Q4 quadrant showed that celastrol promoted ovarian cancer cell apoptosis after the administration of concentrations ranging from 100 nM to 1 µM (100, 500, and 1,000 nM). **(B)** WB analysis showed that celastrol increased BAX and decreased Bcl-2 ranging from 100 nM to 1 µM.

Acell apoptosis kit was used to measure the apoptosis of A2780 cells. A2780 cells were treated with different concentrations of celastrol for 24 h. Celastrol prompted the apoptosis of ovarian cancer cells in a dose-dependent manner after the administration of concentrations ranging from 100 nM to 1 µM (100, 500, and 1,000 nM) (Figure 8). In addition, we detected the expression of apoptosis-related proteins, including BAX and Bcl-2. The results showed that celastrol increased BAX expression while decreasing Bcl-2 expression in a dose-dependent manner.

**FIGURE 9:**
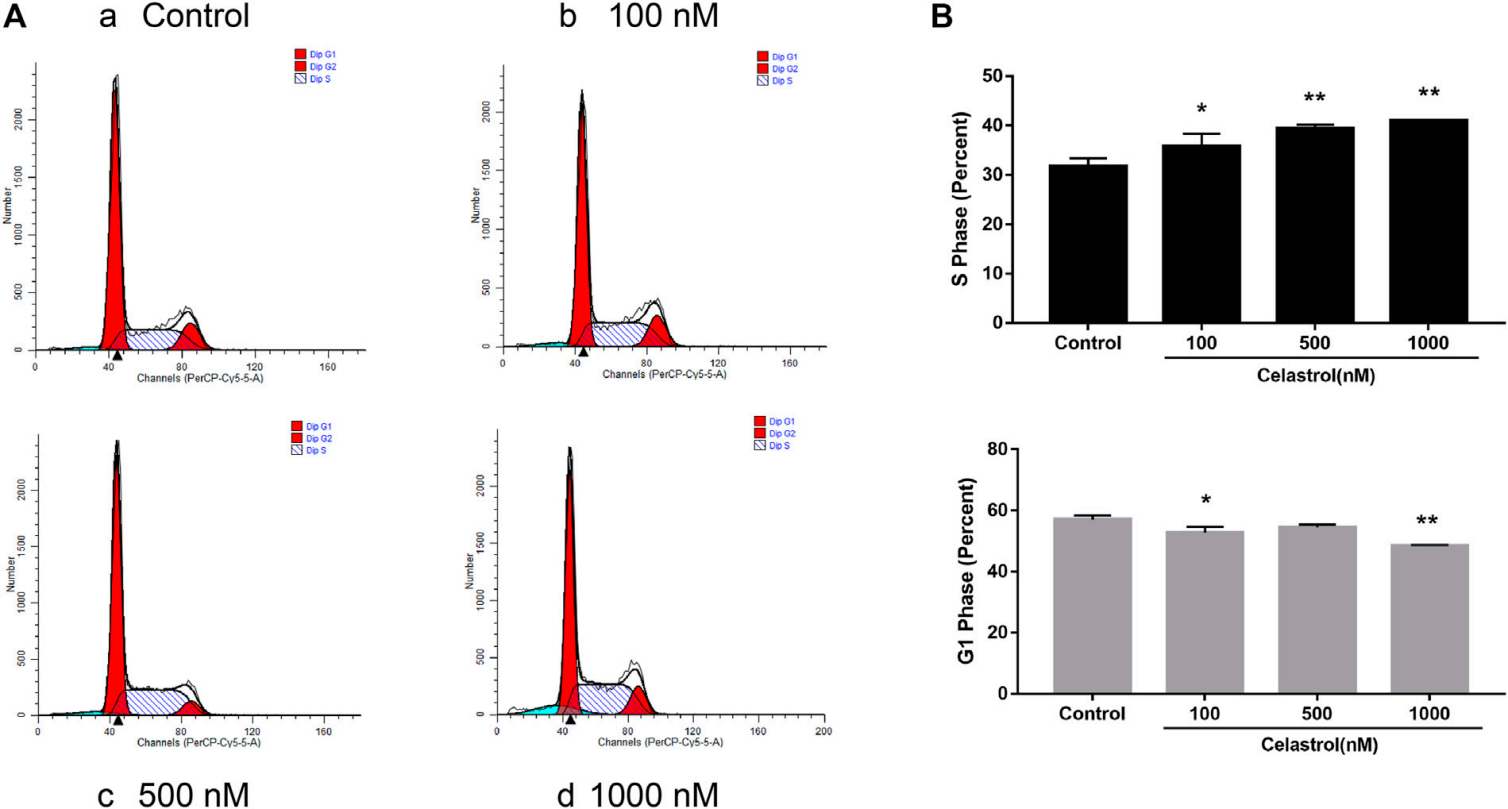
Effect of celastrol on cell cycle in ovarian cancer. After treatment with celastrol, the proportion of cells in S phase was increased compared with control group (from 31.73 ± 1.6% to 40.98 ± 0.02%), while the proportion of G1 phase cells were reduced (from 57.03 ± 1.34% to 48.59 ± 0.19%). Compared with the control group, *p < 0.05, **p < 0.01.

Furthermore, we analyzed the effect of celastrol on the cell cycle. We found that celastrol could increase the percentage of cells in the S phase in a dose-dependent manner (Figure 9). The percentage of cells in the G1 population was decreased after celastrol treatment. The results suggested that celastrol caused the S phase cell cycle arrest in ovarian cancer cells.

### Celastrol Inhibited Core Target Expression in Ovarian Cancer Cells

**FIGURE 10:**
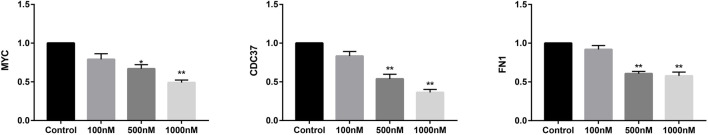
Celastrol inhibited core target expression in ovarian cancer cells. Quantitative RT-PCR analysis of MYC, CDC37, and FN1 mRNA expression. Compared with the control group, *p < 0.05, **p < 0.01.

**FIGURE 11:**
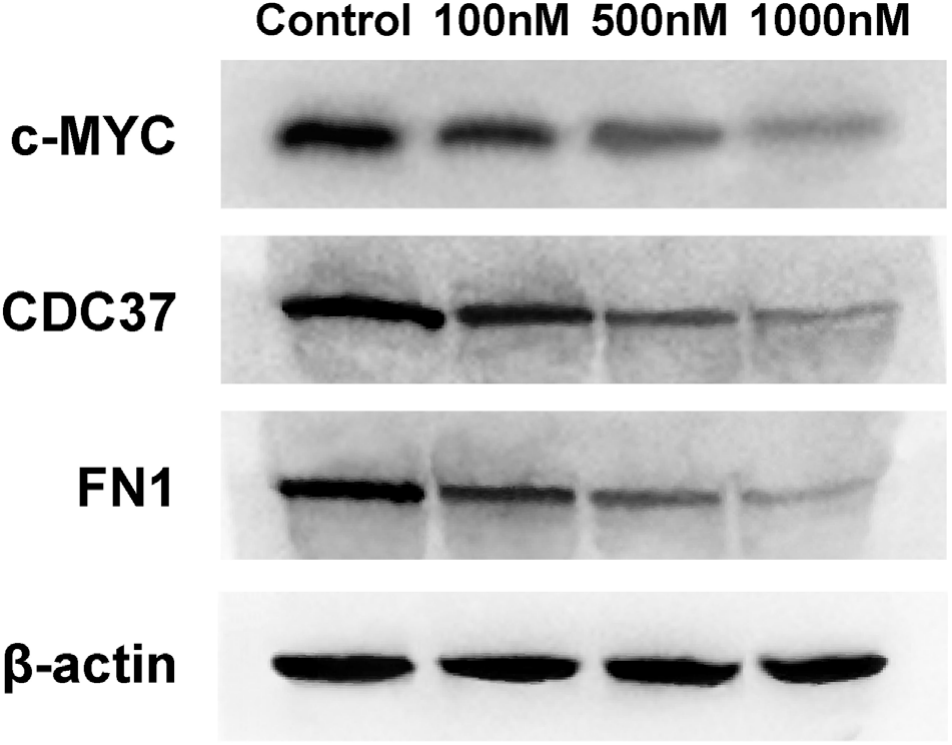
Celastrol inhibited core target expression in ovarian cancer cells. Western blot analysis of c-MYC, CDC37, and FN1 protein expression.

The expression of core targets, including c-MYC, CDC37, and FN1, was evaluated using RT-PCR and WB. As shown in Figure 10, celastrol downregulated the mRNA expression of MYC, CDC37, and FN1 ranging from 100 nM to 1 µM (100, 500, and 1,000 nM). Figure 11 shows that celastrol reduced the levels of the c-MYC, CDC37, and FN1 proteins ranging from 100 nM to 1 µM (100, 500, and 1,000 nM). Thus, celastrol regulated the expression of core targets in ovarian cancer cells.

### Celastrol Inhibited Related Inflammatory Pathways

**FIGURE 12:**
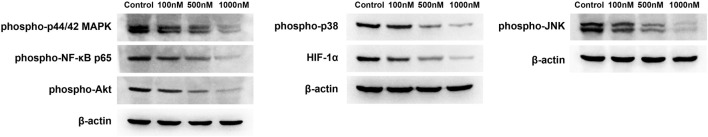
Celastrol inhibited inflammatory pathways. The levels of phospho-p44/42 MAPK, phospho-NF-κB p65, and phospho-Akt were reduced by celastrol ranging from 100 nM to 1 µM. The levels of phospho-JNK, phospho-P38, and HIF-1α were reduced by celastrol ranging from 100 nM to 1 µM.

In the present study, we used WB to measure the levels of inflammatory proteins in specific inflammatory signaling pathways. We found that celastrol reduced the levels of phospho-p44/42 MAPK (Erk1/2). In addition, celastrol reduced phospho-NF-κB p65 and phospho-Akt levels (Figure 12). We also detected the expression of phospho-JNK and phospho-P38. The results showed that celastrol inhibited the expression of phospho-JNK and phospho-P38 in a dose-dependent manner in ovarian cancer cells (Figure 12). Furthermore, we measured the expression of HIF-1α in the HIF-1 signaling pathway. As shown in Figure 12, celastrol inhibited the expression of HIF-1α in a dose dependent manner.

## Discussion

Ovarian cancer is considered one of the most common causes of cancer-related death among women (Gupta et al., 2019). In the United States, more than 20,000 people are estimated to be diagnosed with ovarian cancer, and more than 10,000 people die from this condition annually. Ovarian cancer is difficult to detect in the early stages because patients might not present symptoms. Ovarian cancer is difficult to treat due to delayed detection (Chandra et al., 2019). Celastrol is one of the most promising bioactive compounds derived from TwHF. It regulates the functions of immune cells and inflammatory cytokines to treat inflammatory diseases (Ng et al., 2019). In addition, celastrol has been reported to be a useful treatment for various types of cancer. Previous studies have discovered the mechanism of celastrol against various kinds of cancer. Several processes are involved in the functions of celastrol (Yadav et al., 2018). However, previous studies have not provided a panoramic picture of the function of celastrol in the treatment of ovarian cancer.

We wanted to investigate the function underlying the therapeutic effect of celastrol on ovarian cancer. Network pharmacology was used to determine the core PPI network and related mechanism. Molecular docking was performed to discover the binding affinity between celastrol and key molecules related to ovarian cancer. We found that celastrol had 29 targets and ovarian cancer had 471 targets. The core PPI network contained 163 nodes. The BPs identified in the GO analysis were enriched in telomere maintenance, cellular response to DNA damage stimulus, DNA recombination, cell proliferation, I-kappaB kinase/NF-kappaB signaling, apoptotic process, and other processes. The KEGG analysis indicated that the pathways were enriched in the cell cycle, viral carcinogenesis, pathways in cancer, proteoglycans in cancer, PI3K-Akt signaling pathway, MAPK signaling pathway, HTLV-I infection, HIF-1 signaling pathway, and other pathways. The three core proteins in ovarian cancer that interacted with celastrol were MYC, CDC37, and FN1.

MYC is a family of proto-oncogenes and regulatory genes that encode transcription factors (Casey et al., 2018). MYC is often persistently expressed in cancer, leading to the increased expression of genes involved in cell proliferation (Stine et al., 2015). The activation of MYC is considered a biomarker of cancer initiation and maintenance (Gabay et al., 2014). In the pathogenesis of cancer, MYC induces cancer growth by modulating cellular proliferation, protein synthesis, DNA replication, cellular metabolism, and other processes. MYC is an initiator of tumorigenesis, and MYC overexpression enforces DNA strand breaks, increases oxidative damage, and inhibits double-stranded DNA repair (Gray et al., 2008). In addition, MYC coordinates with other genes to induce tumorigenesis. MYC, along with other genes such as BCL-2, p53, and p19ARF, changes the status of apoptosis and proliferation arrest to induce malignant transformation. Based on the GO results reported in our study, the related BPs also included a cellular response to DNA damage stimulus, DNA recombination, cell proliferation, and apoptotic process. The KEGG results indicated that the cell cycle was involved in the function of celastrol. In this study, we found that celastrol could induce apoptosis and cell cycle arrest in ovarian cancer cells. Due to the important role of MYC in tumorigenesis, MYC targeting is a useful strategy in the treatment of cancer. In our study, celastrol targeted MYC in ovarian cancer. In the interaction of MYC with celastrol, the lowest Vina score was −7.1, while the cavity size was 65 Å^3^. We considered that celastrol might directly interact with MYC to regulate cell proliferation, DNA repair and replication, and apoptosis in ovarian cancer cells.

**FIGURE 13:**
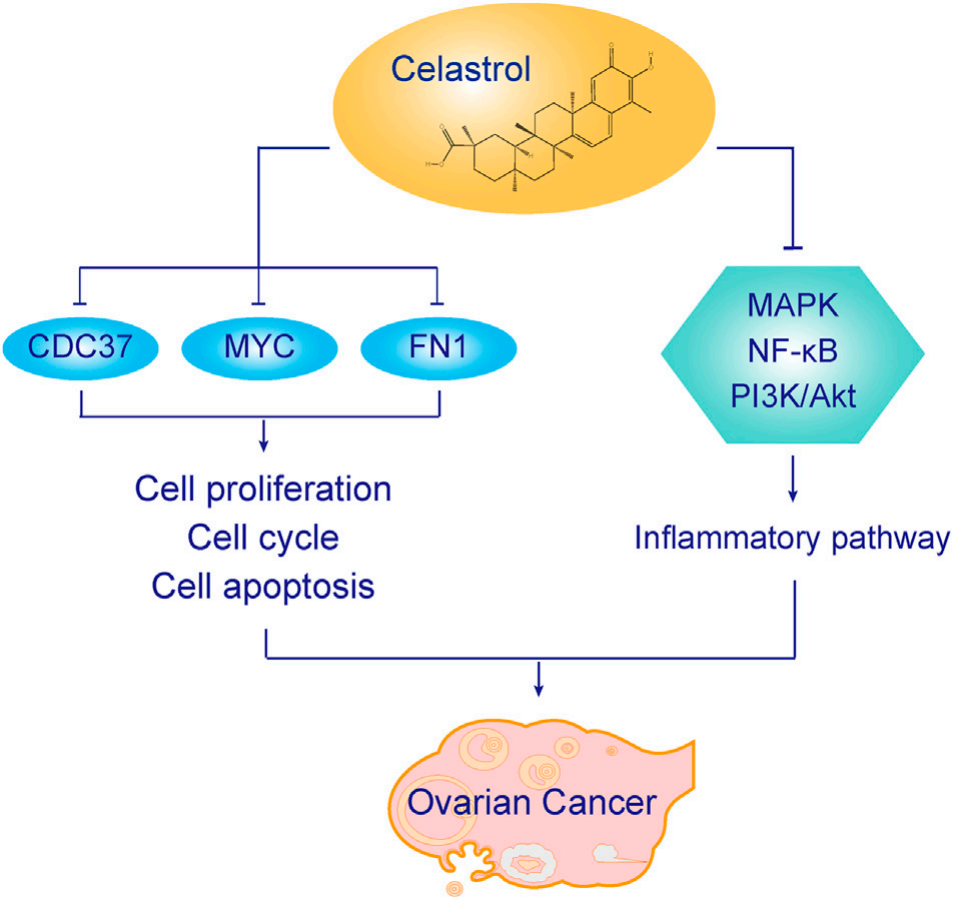
The mechanism underlying the therapeutic effect of celastrol on ovarian cancer.

In the present study, celastrol also directly interacted with CDC37 and FN1. CDC37 is an oncogene regarded as a molecular chaperone (Li et al., 2018). It induces carcinogenesis by regulating oncogenic kinases. CDC37 interacts with HSP90 to accelerate cell proliferation (Dimova et al., 2006). CDC37 is also a target of cancer therapy. In our study, celastrol directly interacted with CDC37 to treat ovarian cancer. The GO analysis indicated that the enriched biological process was cell proliferation. The KEGG analysis showed that the enriched pathways were related to the cell cycle. Thus, celastrol might alter the ovarian cancer cell proliferation and cell cycle by interacting with CDC37. FN1 is involved in the pathogenesis of various tumors. The KEGG analysis indicated that the enriched pathways included the PI3K/Akt pathway. Therefore, celastrol might modulate the PI3K/Akt pathway in ovarian cancer by interacting with FN1. The mechanism by which celastrol inhibits ovarian cancer is shown in Figure 13.

In ovarian cancer, c-MYC, CDC37, and FN1 play essential roles in the initiation and progress of ovarian cancer. First, c-MYC deregulation in ovarian cancer is frequent. c-MYC amplification is established in more than 30% of endometrioid and mixed epithelial ovarian cancers (Du et al., 2018). In addition, c-MYC had a higher frequency of copy-number variations in recurrent patients with ovarian cancer (Wang et al., 1999). c-MYC was correlated with poor survival of ovarian cancer patients (Yoshida, 2018). In the pathogenesis of cancer, MYC induces cancer growth related to cellular proliferation, protein synthesis, DNA replication, cellular metabolism, etc. MYC is the initiator of tumorigenesis, and MYC overexpression enforces DNA breaks and inhibits double-stranded DNA repair (Jiménez-Sánchez et al., 2020). After analyzing the heterogeneity degree of the tumor microenvironment (TME) in ovarian cancer, Jimenez-Sanchez et al. found that MYC amplification was correlated with immune cell exclusion in the TME (Cai et al., 2018). High MYC expression is an independent factor of tumor proliferation in ovarian cancer. Overexpression of MYC targets is cancer-derived and induced by copy number alteration. MYC targets were positively correlated with tumor cellularity. Thus, we inferred that celastrol could regulate c-MYC to alter the tumor microenvironment in dealing with ovarian cancer. Celastrol could interact with c-MYC to inhibit cell proliferation and induce apoptosis in ovarian cancer. FN1 is involved in the pathogenesis of various tumors. Previous studies demonstrated that FN1 was related to the invasion and migration of ovarian cancer cells. Downregulation of FN1 could increase the expression of the proapoptotic protein Bax and decrease the expression of the antiapoptotic protein Bcl-2 in cancer cells (Bao et al., 2021). Online clinical data also showed that ovarian cancer patients had high FN1 expression levels compared with normal people (Kujawa et al., 2020). FN1 was an independent prognostic factor in the overall survival of ovarian cancer patients (Yousif, 2014). In ovarian cancer, FN1 induces invasion and migration by upregulating the PI3K/Akt pathway (Wang et al., 2019). In this study, we found that celastrol could inhibit the expression of FN1. Thus, we inferred that celastrol could inhibit the invasion and migration of ovarian cancer by downregulating the expression of FN1. CDC37 was correlated with cell proliferation in cancer cells (Zhu et al., 2018). In cancer cells, CDC37 could prolong cell survival by activating the CDK4 signaling pathway (Wang et al., 2015). Suppressing CDC37 expression could inhibit cell growth and cell cycle progression (Ono et al., 2020). In addition, CDC37 plays a vital role in the epithelial–mesenchymal transition (EMT) of cancer progression (Wagner and Nebreda, 2009). In this study, we found that celastrol could downregulate the expression of CDC37. Therefore, we inferred that celastrol might inhibit cancer cell growth and the cell cycle by downregulating CDC37 expression.

In addition to MYC, CDC37, and FN1, we discovered that celastrol regulated inflammatory pathways in ovarian cancer. The KEGG analysis showed that the enriched inflammatory pathways were the MAPK signaling pathway, HIF-1 signaling pathway, and NF-κB pathway. The MAPK signaling pathway plays an important role in cancer cell invasion, migration, and drug resistance (Roy et al., 2018). This pathway modulates DNA damage and metabolic stress in cancer cells (Macklin et al., 2017). MAPK signaling cascade activation increases the expression of inflammatory cytokines such as TNF-α and interleukin-8 in cancer. These inflammatory cytokines are related to the growth and apoptosis of ovarian cancer cells. Thus, we inferred that celastrol might exert anticancer effects by inhibiting the MAPK signaling pathway. The HIF-1 signaling pathway is considered a cancer drug target related to angiogenesis, hypoxic stress, cancer cell growth, and metastasis (39). In the present study, celastrol regulated the MAPK, HIF-1, and NF-κB signaling pathways to modulate the inflammatory status of ovarian cancer.

## Conclusion

In this study, we used *in vitro* and *in vivo* experiments to discover the mechanism by which celastrol treats ovarian cancer. Network pharmacology was used to determine the core PPI network and related mechanism. Molecular docking was used to discover the binding affinity between celastrol and key molecules related to ovarian cancer. The core PPI network contained 163 nodes. The BPs identified in the GO analysis were enriched in telomere maintenance, cellular response to DNA damage stimulus, DNA recombination, cell proliferation, I-kappaB kinase/NF-kappaB signaling, apoptotic process, and other processes. The KEGG analysis indicated that the enriched pathways were related to the cell cycle, viral carcinogenesis, pathways in cancer, proteoglycans in cancer, PI3K-Akt signaling pathway, MAPK signaling pathway, HTLV-I infection, HIF-1 signaling pathway, and other pathways. The three core proteins in ovarian cancer that interacted with celastrol were MYC, CDC37, and FN1. Celastrol directly interacted with these targets to exert antitumor effects. Celastrol directly interacted with MYC to regulate cell proliferation, DNA repair and replication, and apoptosis in ovarian cancer cells. Celastrol altered the proliferation and cell cycle of ovarian cancer cells by interacting with CDC37. Celastrol might modulate the PI3K/Akt pathway in ovarian cancer by interacting with FN1. Furthermore, celastrol potentially regulates inflammatory pathways, including the MAPK signaling pathway, HIF-1 signaling pathway, and NF-κB signaling pathway, in ovarian cancer. Based on the results of this study, we hope to raise a perspective and valid theoretical foundation for the use of celastrol to treat ovarian cancer in future research.

## Data Availability

The original contributions presented in the study are included in the article, further inquiries can be directed to the corresponding authors.
